# First-Principles Calculations, Experimental Study, and Thermodynamic Modeling of the Al-Co-Cr System

**DOI:** 10.1371/journal.pone.0121386

**Published:** 2015-04-13

**Authors:** Xuan L. Liu, Thomas Gheno, Bonnie B. Lindahl, Greta Lindwall, Brian Gleeson, Zi-Kui Liu

**Affiliations:** 1 Department of Materials Science and Engineering, The Pennsylvania State University, University Park, Pennsylvania, 16802, United States of America; 2 Department of Mechanical Engineering and Materials Science, University of Pittsburgh, Pittsburgh, Pennsylvania, 15261, United States of America; 3 Department of Materials Science and Engineering, KTH Royal Institute of Technology, SE-100 44, Stockholm, Sweden; University of Calgary, CANADA

## Abstract

The phase relations and thermodynamic properties of the condensed Al-Co-Cr ternary alloy system are investigated using first-principles calculations based on density functional theory (DFT) and phase-equilibria experiments that led to X-ray diffraction (XRD) and electron probe micro-analysis (EPMA) measurements. A thermodynamic description is developed by means of the calculations of phase diagrams (CALPHAD) method using experimental and computational data from the present work and the literature. Emphasis is placed on modeling the bcc-A2, B2, fcc-γ, and tetragonal-σ phases in the temperature range of 1173 to 1623 K. Liquid, bcc-A2 and fcc-γ phases are modeled using substitutional solution descriptions. First-principles special quasirandom structures (SQS) calculations predict a large bcc-A2 (disordered)/B2 (ordered) miscibility gap, in agreement with experiments. A partitioning model is then used for the A2/B2 phase to effectively describe the order-disorder transitions. The critically assessed thermodynamic description describes all phase equilibria data well. A2/B2 transitions are also shown to agree well with previous experimental findings.

## Introduction

Nickel-base superalloys used in the hot sections of gas turbines require corrosion-resistant coatings to withstand the harsh thermo-chemical conditions prevailing in these combustion environments [1,2]. Overlay MCrAlY (M = Ni, Co or NiCo) coatings are widely used for this purpose, either solely or in conjunction with a thermally insulating ceramic topcoat, most commonly yttria-stabilized zirconia, to comprise a thermal barrier coating (TBC) system [3–6]. During service, microstructural evolution of the MCrAlY layer arises as a result of temperature variations, reactions with the environment such as selective oxidation removing Al from the subsurface, or interdiffusion with the substrate material, driven by differences in chemical potentials. A good knowledge of the alloy thermodynamics is critical to understanding and predicting these evolutionary processes, which in turn affect the corrosion resistance and mechanical properties of the system.

Computational design of effective MCrAl-based coating compositions necessitates a thermodynamic description methodology which can predict phase compositions and fractions at the temperatures of interest. One such method is CALPHAD, which has been used extensively and successfully in the thermodynamic modeling of many multi-component systems including Ni-base superalloys [7,8]. The CALPHAD approach parameterizes the Gibbs energy functions of all phases in unary, binary and ternary systems using computational and experimental data on phase equilibria and thermochemistry. The parameters are entered in a database and extrapolations to multi-component systems can be made. Limitations are known to exist in the current description of the quaternary Al-Co-Cr-Ni system for Co contents in excess of 20 wt. % [9,10] which are common in practice [2,11,12]. While assessment of the Al-Cr-Ni system has been well documented [7], the thermodynamic properties of the Al-Co-Cr system have not been adequately assessed.

Phases important to coating systems are B2 (β, $Pm\bar{3}m$, simple cubic type) and fcc-A1 (γ, $Fm\bar{3}m$, disordered f.c.c.). However, bcc-A2 (α, $Im\bar{3}m$, disordered b.c.c.), hcp-A3 (ε, *P*6_3_ / *mmc*, disordered h.c.p.) and sigma (σ, *P*4_2_ / *mnm*, Frank-Kasper) must also be modeled to allow for a complete description. One important feature of the Al-Co-Cr system is the A2/B2 second-order transformation at temperatures above 1373 K [13]. The present study aims to construct a thermodynamic model accounting for the A2/B2 ordering phenomenon, where atoms take on distinct lattice sites in B2. The two phases have vastly different compositions at low temperatures, but merge to similar compositions at higher temperatures which has to be accounted for in the thermodynamic model.

As phase equilibrium data are scarce, especially in the important B2+γ+σ region, experiments are conducted in this study at 1173, 1273, and 1373 K to aid the thermodynamic modeling. Additionally, first-principles calculations based on density functional (DFT) theory are incorporated to supplement the lack of experimentally measured thermochemical data in the ternary system [14] as well as to study the A2+B2 miscibility gap that is prevalent over the entire phase diagram. Ultimately, this system will be used in the construction of a multicomponent Al-Co-Cr-Ni-Y database. As a result, this database needs to be compatible with previously modeled Al-Co-Ni [15] and Al-Cr-Ni [7] systems.

The Al-Co thermodynamic model assessed by Dupin and Ansara [16] is adopted in the present work. This will allow compatibility with Dupin’s [15] model of the Al-Co-Ni system. The Co-Cr system modeled by Oikawa et al. [17] is found to successfully capture experimental activity [18], enthalpy [18] and phase boundary data [17], which are crucial toward the extension to the Al-Co-Cr ternary, and is therefore adopted here. It also allows compatibility with the modeling of the Co-Cr-Ni system by Yang et al. [19], which would enable modeling of the quaternary Al-Co-Cr-Ni system. In Oikawa et al.’s [17] treatment of the Co-Cr system, no indication of B2 ordering was reported, hence the B2 phase will be modeled as a metastable phase with data from first-principles calculations. Saunders' [20] assessment of the Al-Cr system, used in COST507, is used here. Its description of the composition range of some intermetallics is not complete, but this will be shown to have no impact on the Al-Co-Cr system at the temperatures of interest in this study. Although XRD data from Helander and Tolochko [21] indicate a possible B2 ordering, no A2 to B2 second-order transition has been conclusively established in the Al-Cr system. Following Dupin et al. [7] who noted that a A2/B2 transition would modify the A2-AlCr_2_ phase boundary and require additional experimental data for proper assessment, the B2-AlCr phase is not included here.

Ishikawa et al. [13] investigated the Al-Co-Cr system in the temperature range 1273–1623 K using multi-phase alloys and diffusion couples. Isotherms at 1573 and 1623 K showed complete dissolution of σ and a particularly large composition range for the B2 phase. The experimental work also provided compositions for the A2/B2 order-disorder transition at 1473 K and above, which are important to the present modeling. Previous models are combined with ternary data from the literature and this work to produce the overall thermodynamic description.

## Materials and Methods

### Experimental procedures

Ingots of nominal compositions (at. %) Co-20Cr-20Al-0.4Y (# A4), Co-26Cr-11Al-0.1Y (A5), Co-35Cr-6Al-0.1Y (A6) and Co-35Cr-11Al-0.1Y (A7) were prepared by arc-melting and drop-cast in a chamber evacuated and back-filled with 0.5 atm of argon. Specimens were cut to approximate dimensions of 10 x 10 x 1 mm, vacuum-encapsulated in quartz capsules, homogenized for 48 h at 1423 K in a tube furnace, and slowly brought to equilibration temperature. Equilibration treatments were conducted at 1173, 1273 and 1373 K for 500, 200 and 100 h, respectively, followed by water quenching to retain the equilibrated microstructures. Phase constitutions were studied by X-ray diffraction (XRD) with a PANalytical Empyrean instrument, using a Co radiation source (K_α1_ = 1.789 Å).

Polished sections of the heat-treated alloys were prepared by standard metallographic procedures. Phase compositions were determined by electron probe micro-analysis (EPMA) using a JEOL JXA-8530F field emission gun instrument. For each element, measured intensities were converted to concentrations by interpolation via a calibration curve built using a series of standards of known compositions (chemical analysis by inductively coupled plasma mass spectrometry). The probe size used during measurements was about 1 μm, and the alloy microstructures were sufficiently coarse for each phase to be analyzed individually.

### CALPHAD thermodynamic models

#### Solution phases: bcc, fcc (γ), hcp (ε), and liquid

In the present work, the Gibbs energies of γ, ε, and liquid solution phases are modeled using the following equation based on a single sublattice,

$$G_{m}^{\Phi }=\sum x_{i}{\;}^{o}G_{i}^{\Phi }+RT\;\sum x_{i}lnx_{i}+{\;}^{XS}G_{m}^{\Phi }+RT{\;}^{Magnetic}G_{m}^{\Phi }$$ Eq. 1

where ${}^{o}G_{i}^{\Phi }$ represents the molar Gibbs energy of the pure elements, *i* = Al, Co, Cr, with structure Φ taken from the SGTE Unary PURE4 database [22], and the other symbols have their usual meanings. In Eq. 1, the first term represents the physical mixing of the elements and the second term is the contribution from the ideal entropy of mixing. The excess term, ${}^{XS}G_{m}^{\Phi }$, is modeled using a Redlich-Kister polynomial [23] to represent the non-ideal interactions between Al, Co and Cr, as shown below:

$$\begin{array}{l} {}^{XS}G_{m}^{\Phi }=\sum_{i}\sum_{j>i}x_{i}x_{j}\sum_{v=0}{}^{v}L_{i,j}^{\Phi }\left(x_{i}-x_{j}\right)\\ +\sum_{i}\sum_{j>i}\sum_{k>j}x_{i}x_{j}x_{k}\left[{}^{1}L_{i,j,k}^{\Phi }\left(x_{i}+\delta_{i,j,k}\right)+{}^{2}L_{i,j,k}^{\Phi }\left(x_{j}+\delta_{i,j,k}\right)+{}^{3}L_{i,j,k}^{\Phi }\left(x_{k}+\delta_{i,j,k}\right)\right] \end{array}$$ Eq. 2

Where *δ*_*i*,*j*,*k*_ = (1−*x*_*i*_−*x*_*j*_−*x*_*k*_) / 3. The binary and ternary interaction terms, ${}^{v}L_{i,j}^{\Phi }$ and ${}^{v}L_{i,j,k}^{\Phi }$, are expressed in the form *A* + *B*×*T* where *A* and *B* are the model parameters to be evaluated. Due to the ferromagnetic nature of Co and the antiferromagnetic nature of Cr, ${}^{Magnetic}G_{m}^{\Phi }$ is included to capture magnetic contributions to the Gibbs energy [24,25]. Hillert and Jarl [24] expressed the magnetic contribution as:

$${}^{Magnetic}G_{m}^{\Phi }=RTln\left(\beta +1\right)f\left(\tau \right)$$ Eq. 3

where *β* represents the average magnetic moment and the function *f*(*τ*) is related to the Curie temperature (*T*_*C*_):

$$\begin{array}{l} f\left(\tau \right)=1-\frac{1}{A}\left[\frac{79\tau^{-1}}{140p}+\frac{474}{497}\left(\frac{1}{p}-1\right)\left(\frac{\tau^{3}}{6}+\frac{\tau^{9}}{135}+\frac{\tau^{15}}{600}\right)\right],if\;\tau =\frac{T}{T_{C}}\leq 1\\ f\left(\tau \right)=-\frac{1}{A}\left(\frac{\tau^{-5}}{10}+\frac{\tau^{-15}}{315}+\frac{\tau^{-25}}{1500}\right),if\;\tau =\frac{T}{T_{C}}\geq 1\\ A=\frac{518}{1125}+\frac{11,692}{15,975}\left(\frac{1}{p}-1\right) \end{array}$$ Eq. 4

The constant *p* takes on values of 0.28 for fcc and hcp metals and 0.4 for bcc metals.

To account for the A2/B2 order-disorder transition, the bcc phase is modeled using the partitioning model, which treats the ordered and disordered components separately [26]. The disordered component of A2 is described using Eq. 1, and the model for the ordered component is described in the next section. Additionally, triple-defect mechanisms are especially important when considering site ordering in B2-aluminide-containing systems such as Al-Co [16], Al-Fe [27] or Al-Ni [7]. To account for this phenomenon [29] when the A2 phase is combined with the B2 phase, vacancies have been introduced in the substitutional sublattice of the A2 phase model as in Ref. [26]. Ordering of the bcc phase will be described in the following section.

### Intermetallic phases: B2 and σ

The two ordered phases, B2 and σ, are described using sublattice models based on the compound energy formalism (CEF) [28]. In order to describe the A2/B2 ordering for the bcc phase, both A2 and B2 are modeled using a single Gibbs energy function where the ordered part (B2) is described by a sublattice formula (Al,Co,Cr,Va)_1_(Al,Co,Cr,Va)_1_ and the disordered part (A2) by (Al,Co,Cr,Va)_1_. Specifically, B2 is appended to the already existing A2 model. Then, as implemented in Thermo-Calc, free energy minimization determines whether A2 and/or B2 are stable depending on the input conditions. In addition, a set of parameterization constraints derived by Dupin and Ansara [26] are used to partition the ordered and disordered parts of the A2/B2 Gibbs energy to allow independent evaluations. The B2 phase contains six stoichiometric compounds, or "end-members" (excluding vacancies) which represent the reference states generated when pure components fully occupy a sublattice; i.e. B2-(Al)(Al), B2-(Al)(Co), B2-(Al)(Cr), B2-(Co)(Cr), B2-(Co)(Co), and B2-(Cr)(Cr). The end-members of the B2 phase in the present system all come from binary systems and as a result, their formation energies are fixed by the previous Al-Co and Al-Cr binary assessments in this model; B2 is not included in the Co-Cr assessment by Oikawa et al. [17] and is therefore modeled in the present work.

The partitioning model describes the A2 and B2 with one single Gibbs energy function and the same sublattice model, (Al,Co,Cr,Va)_1_(Al,Co,Cr,Va)_1_. As a result, the Gibbs energy of A2 combined with B2 is described as:

$$G_{m}^{\alpha /\beta }=G_{m}^{\beta }\left(x_{i}\right)+\Delta G_{m}^{order}\left(y_{i}^{\left(s\right)}\right)$$ Eq. 5

to include contributions from the disordered solution, $G_{m}^{\beta }\left(x_{i}\right)$, in A2 as well as from the ordered B2 itself, $\Delta G_{m}^{order}\left(y_{i}^{\left(s\right)}\right)$. Here, $\Delta G_{m}^{order}\left(y_{i}^{\left(s\right)}\right)$ can be separated into two terms, and the total Gibbs energy of A2 and B2 becomes:

$$G_{m}^{\alpha /\beta }=G_{m}^{\alpha }\left(x_{i}\right)+G_{m}^{\beta \left(O\right)}\left(y_{i}^{\left(s\right)}\right)-G_{m}^{\beta \left(O\right)}\left(y_{i}^{\left(s\right)}=x_{i}\right)$$ Eq. 6

where $y_{i}^{\left(s\right)}$ denotes the site fraction of element *i* on sublattice *s*. It can be seen that the ordering independent term, $G_{m}^{\alpha }\left(x_{i}\right)$, and the ordering dependent terms $G_{m}^{\beta \left(O\right)}\left(y_{i}^{\left(s\right)}\right)$ and $G_{m}^{\beta \left(O\right)}\left(y_{i}^{\left(s\right)}=x_{i}\right)$ are separated in a way to allow each to be modeled independently. The term $G_{m}^{\beta \left(O\right)}\left(y_{i}^{\left(s\right)}\right)$ takes on the partitioned form, as described by Dupin and Ansara [26]:

$$\begin{array}{l} G_{m}^{\beta \left(O\right)}\left(y_{i}^{\left(s\right)}\right)=\sum_{i}\sum_{j}\left(y_{i}^{'}y_{j}^{''}\;{}^{o}G_{i:j}^{\beta \left(O\right)}+y_{j}^{'}y_{i}^{''}\;{}^{o}G_{j:i}^{\beta \left(O\right)}\right)+RT\left[\sum_{i}y_{i}^{'}\ln\left(y_{i}^{'}\right)+\sum_{j}y_{j}^{''}\ln\left(y_{j}^{''}\right)\right]\\ +\sum_{i}\sum_{j}\sum_{k}\left\{y_{i}^{'}y_{j}^{'}y_{k}^{''}\left[\sum_{k}{}^{v}L_{i,j:k}^{\beta \left(O\right)}{\left(y_{i}^{'}-y_{j}^{'}\right)}^{v}\right]\right\}+\sum_{i}\sum_{j}\sum_{k}\left\{y_{k}^{'}y_{i}^{''}y_{j}^{''}\left[\sum_{k}{}^{v}L_{k:i,j}^{\beta \left(O\right)}{\left(y_{i}^{''}-y_{j}^{''}\right)}^{v}\right]\right\}\\ +\sum_{i}\sum_{j}\sum_{k}\sum_{l}y_{i}^{'}y_{j}^{'}y_{k}^{''}y_{l}^{''}\;{}^{v}L_{i,j:k,l}^{\beta \left(O\right)}+\sum_{i}\sum_{j}\sum_{k}\sum_{l}\left(y_{i}^{'}y_{j}^{'}y_{k}^{'}y_{l}^{''}\;{}^{v}L_{i,j,k:l}^{\beta \left(O\right)}+y_{i}^{''}y_{j}^{''}y_{k}^{''}y_{l}^{'}\;{}^{v}L_{l:i,j,k}^{\beta \left(O\right)}\right) \end{array}$$ Eq. 7

When the phase becomes disordered, i.e. $y_{i}^{'}=y_{i}^{''}=x_{i}$, $G_{m}^{\beta \left(O\right)}\left(y_{i}^{\left(s\right)}\right)-G_{m}^{\beta \left(O\right)}\left(y_{i}^{\left(s\right)}=x_{i}\right)$ equals zero and eliminates the ordering energy contribution. The equivalence of the CEF and partitioned models is obtained using $2G_{m}^{Partition}\;\left(y_{i}^{\left(s\right)}\right)=G_{m}^{CEF}\;\left(y_{i}^{\left(s\right)}\right)$. In Eq. 7, ${}^{o}G_{i:j}^{\beta \left(O\right)}$ represents the Gibbs energy of a B2 end-member expressed by Eq. 10 with *i* and *j* in the first and second sublattices, respectively. $L_{i:j,k}^{\beta \left(O\right)}$ is the interaction term between end-members of *i*:*j* and *i*:*k* that takes the form of *A* + *B*×*T* where *A* and *B* are the model parameters to be evaluated in modeling process. Relationships between the interaction parameters used in the CEF and partitioned models exist and are derived by Dupin and Ansara [26]. B2 end-members have both sites equivalent to one another so A2 disordering is possible when those sites have the same disordered occupancy. This crystallographic site equivalency is taken into account with the following expressions [26]:

$${}^{o}G_{i:j}^{\beta }={}^{o}G_{j:i}^{\beta },\;{}^{v}L_{i,j:k}^{\beta }={}^{v}L_{k:i,j}^{\beta },\;{}^{v}L_{i,j:k,l}^{\beta }={}^{v}L_{k,l:i,j}^{\beta },\;\text{and}\;{}^{v}L_{i,j,k:l}^{\beta }={}^{v}L_{l:i,j,k}^{\beta }$$ Eq. 8

As adopted from the Co-Cr binary [17], σ is modeled with the sublattice formulation (Al,Co)_8_(Al,Co,Cr)_18_(Cr)_4_. The phase σ contains σ-(Al)_8_(Al)_18_(Cr)_4_, σ-(Al)_8_(Cr)_18_(Cr)_4_, σ-(Co)_8_(Co)_18_(Cr)_4_, σ-(Co)_8_(Cr)_18_(Cr)_4_ binary and σ-(Al)_8_(Co)_18_(Cr)_4_, σ-(Co)_8_(Al)_18_(Cr)_4_ ternary end-members. To extend the solubility of σ from the binary Co-Cr system, Al must be introduced while keeping consistency with the original model for σ. Joubert [29] suggested that Al should only be allowed to mix in the first and second sublattices given its size and electronic characteristics. In accordance with this, a model described as (Al,Co)_8_(Al,Co,Cr)_18_(Cr)_4_ is established. The Gibbs energy of σ in per mole of formula has the form,

$$\begin{array}{l} G_{m}^{\sigma }=\sum_{i}\sum_{j}y_{i}^{'}y_{j}^{''}\;{}^{o}G_{i:j:Cr}^{\sigma }+RT\left[8\sum_{i}y_{i}^{'}\ln\left(y_{i}^{'}\right)+18\sum_{j}y_{j}^{''}\ln\left(y_{j}^{''}\right)\right]\\ +\sum_{i_{1}}\sum_{i_{2}}\sum_{j}\left\{y_{i_{1}}^{'}y_{i_{2}}^{'}y_{j}^{''}\left[\sum_{k}{}^{k}L_{i_{1},i_{2}:j:Cr}^{\sigma }{\left(y_{i_{1}}^{'}-y_{i_{2}}^{'}\right)}^{k}\right]\right\}\\ +\sum_{i}\sum_{j_{1}}\sum_{j_{2}}\left\{y_{i}^{'}y_{j_{1}}^{''}y_{j_{2}}^{''}\left[\sum_{k}{}^{k}L_{i:j_{1},j_{2}:Cr}^{\sigma }{\left(y_{j_{1}}^{''}-y_{j_{2}}^{''}\right)}^{k}\right]\right\} \end{array}$$ Eq. 9

where $y_{i}^{'}$ and $y_{i}^{''}$ represent the site fractions of *i* in the first and second sublattices, ${}^{o}G_{i:j:Cr}^{\sigma }$ represents the Gibbs energy of particular σ end-members as shown by Eq. 10, and $L_{i:j,k:Cr}^{\sigma }$ is the interaction term between end-members of *i*:*j*:Cr and *i*:*k*:Cr.

The described binary and ternary end-members for B2 and σ are modeled as follows,

$$G_{m}^{Al_{x}Co_{y}Cr_{z}}=x\;{}^{o}G_{Al}^{\text{fcc}}+y\;{}^{o}G_{Co}^{\text{hcp}}+z\;{}^{o}G_{Cr}^{\text{bcc}}+\Delta_{\;f}G^{Al_{x}Co_{y}Cr_{z}}$$ Eq. 10

where $\Delta_{\;f}G^{Al_{x}Co_{y}Cr_{z}}=\Delta_{\;f}H^{Al_{x}Co_{y}Cr_{z}}-T\Delta_{\;f}S^{Al_{x}Co_{y}Cr_{z}}$ and represents the Gibbs energy of formation of a particular B2 or σ end-member with the composition Al_x_Co_y_Cr_z_. The Debye-Grüneisen model is used to predict the enthalpy and entropy of the compounds as a function of temperature from 0 K properties obtained by the DFT calculations. Details for these calculations are presented in the following section. The PARROT module within Thermo-Calc [30] is used to assess all model parameters for each phase in the system using the phase equilibrium data from our experimental measurements and the results by Ishikawa et al. [13] as well as first-principles thermochemical data calculated in the present work. PARROT is a thermodynamic data assessment module which has been developed to fit model parameters to experimental data by a least mean square method [31].

### First-principles methodologies

The Helmholtz energy, *F*(*V*,*T*), of a condensed phase, in terms of the quasiharmonic approach, from first-principles calculations based on DFT is expressed as follows [32,33]:

$$F\left(V,T\right)=E_{0K}\left(V\right)+F_{Vib}\left(V,T\right)+F_{T-el}\left(V,T\right)$$ Eq. 11

In the above expression, *F*_*Vib*_(*V*,*T*) and *F*_*T-el*_(*V*,*T*) represent the temperature-dependent vibrational and thermal-electronic contributions, respectively. In the present work, the Helmholtz energy is taken approximately as the Gibbs energy due to the negligible ambient pressure used in the modeling. The thermal electronic contribution to the Helmholtz energy is estimated based on the electronic density of states and calculated using Fermi-Dirac statistics for metallic systems [32]. *E*_*0K*_(*V*) is the static contribution at 0 K without the zero-point vibrational energy. It is obtained using a four-parameter Birch-Murnaghan (BM4) equation of state (EOS) [32,34]:

$$E_{0K}\left(V\right)=a+bV^{-2/3}+cV^{-4/3}+dV^{-2}$$ Eq. 12

where *a*, *b*, *c*, and *d* are fitting parameters. Energy versus volume (E-V) data used in the fitting are relaxed with respect to ionic positions and cell shape at the given volumes.

Lattice vibrations are modeled with the Debye-Grüneisen model with the benefit of both accuracy and efficiency; the relevant equations have been described in detail in previous publications [35,36]. The scaling factor is implemented to scale the Debye temperature as a consequence of the differences in transverse and longitudinal phonon modes [37]. It has been shown that the scaling factor is highly dependent on the crystal structure [38,39]. This factor can be estimated from elastic constant calculations using DFT and then averaged as an isotropic medium. Elastic constant calculations are implemented in this work for fcc-Al, hcp-Co, bcc-Co, B2, and σ using the method proposed by Shang et al. [40].

In order to predict A2 solution mixing, calculations of the enthalpy of mixing based on special quasirandom structures calculations (SQS) [41] are performed using the 16-atom binary A2 model developed by Jiang et al. [42] and the 32- or 36-atom ternary A2 model developed by Jiang [43]. Additionally, ternary B2 solution mixing calculations are also performed using 8- and 16-atom supercells generated for the isostructural B2-AlNi system by Jiang et al. [44]. SQS calculation procedures for the present work are performed with the method recently implemented by Lieser et al. [45]. The SQS supercells are first relaxed with respect to cell volume only, and then to only cell volume and shape, and finally to cell volume, shape as well as ion positions simultaneously. Radial distribution functions (RDF) of relaxed supercells are compared with the ideal bcc structure after each relaxation step [45]. The structures with the lowest energy that retain the required structural symmetry are used in the present work.

The Vienna *ab-initio* Simulation Package (VASP) [46] is used for spin-polarized DFT calculations due to the ferro- and antiferromagnetic natures of Co and Cr, respectively. Electron-ion interactions are described by the accurate projector augmented-wave (PAW) method [47,48]. The generalized gradient approximation (GGA) as implemented by Perdew, Burke, and Ernzerhof (PBE) [49] is used to describe the electron exchange and correlation. A plane-wave cutoff energy of 400 eV is consistently used to ensure enough basis sets are included, as recommended by the VASP manual [50]. Reciprocal *k*-meshes used for fcc-Al/Co, hcp-Co, bcc-Al/Co/Cr, B2 and σ are 21×21×21, 23×23×12, 17×17×17, 15×15×15, and 6×6×11, respectively. The structures are relaxed by implementing the Methfessel-Paxton method [51] to minimize the forces acting on the atoms. After relaxations, a final calculation using the tetrahedron with Blöchl corrections [52] is applied to ensure an accurate total energy calculation.

## Results and Discussion

### Experimental results

**Fig 1:**
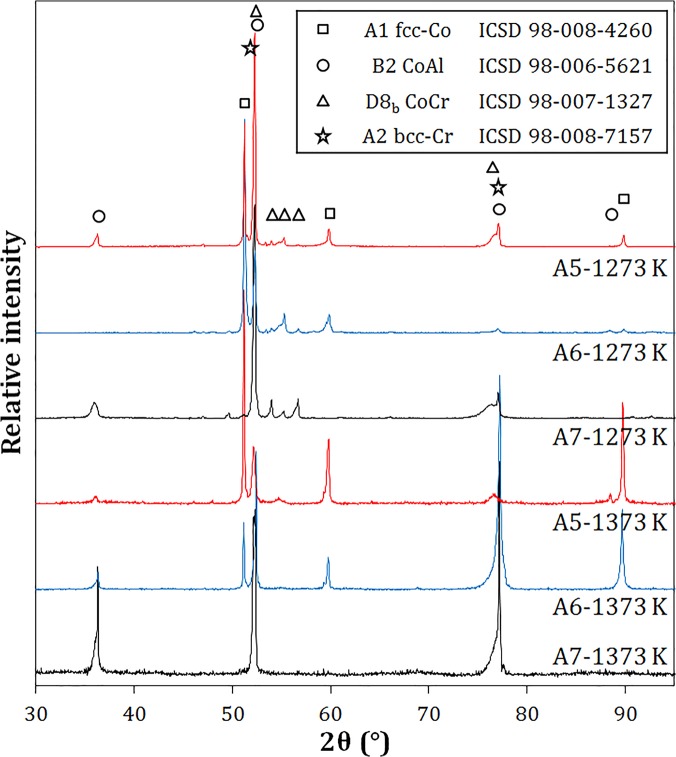
XRD analysis of selected alloy phase constitutions at 1273 K and 1373 K. Note that preferential orientations inherent to cast microstructures were still present after annealing. Specimens were rotated in-plane to ensure that all phases were detected.

**Fig 2:**
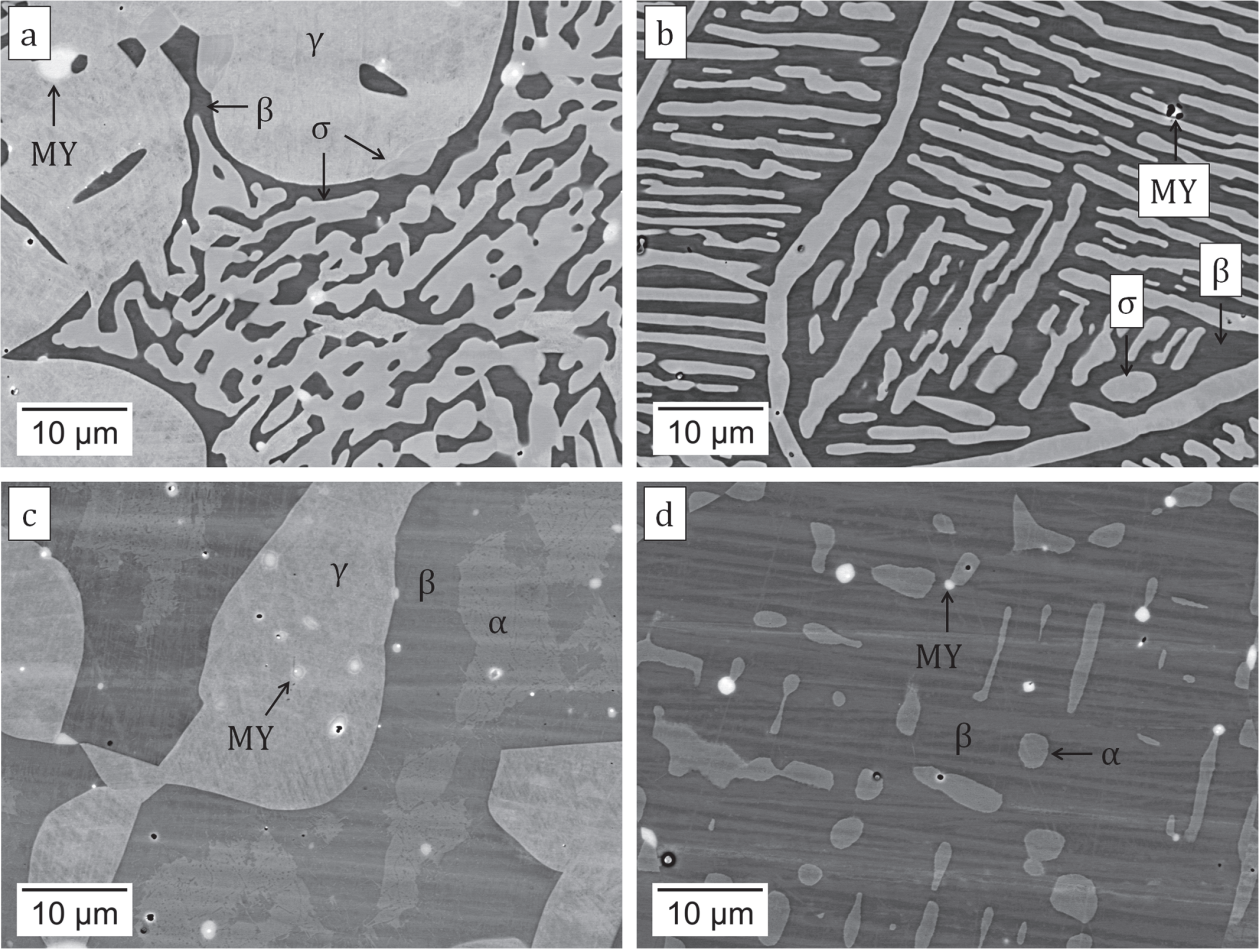
Microstructure of alloys (a,c) A6 and (b,d) A7 equilibrated at (a,b) 1273 K and (c,d) 1373 K. The bright precipitates are Y-containing intermetallics (noted MY).

Alloy phase constitutions were determined through a combination of XRD (selected spectra shown in Fig 1) and phase composition analyses (EPMA results given in Table 1). At 1173 and 1273 K, the alloys A4, A5 and A7 are within the B2-γ-σ three-phase triangle, which is an invariant equilibrium in a three-component system. Small differences measured between the phase compositions are larger than the experimental standard deviation, and may reflect the slight influence of Y on phase equilibria. Selected microstructures observed after equilibration at 1273 and 1373 K are shown in Fig 2. In Fig 2A and 2B, the dark matrix is B2, the two light phases are γ and σ, and the bright precipitates are Y-containing intermetallics (denoted MY). These phases are identified based on their measured compositions and associated XRD results. This phase constitution is typical for equilibrium at 1173 and 1273 K. At 1373 K, the B2 phase exhibits compositions that vary widely between the four alloys studied. These are substantially different from those measured at 1173 and 1273 K, with Al contents as low as 16.2 at.% and Cr contents as high as 36.9 at.%. Nevertheless, XRD confirmed that the ordered B2 structure is maintained at 1373 K. In the absence of σ reflections, it is concluded that σ has been replaced by A2, which dissolves significantly more Al (Table 1). The A2 and B2 phases each have a cubic structure, and their peaks cannot be resolved with the instrument available. Analysis by EPMA (not shown here) indicated that two types of Co-rich yttrides were present, containing ~ 7 at.% and ~ 9 at.% Y, with 18–25 at.% Cr and 9–14 at.% Al. Due to the low volume fraction of these phases, their structures could not be determined by XRD.

**Table 1:** Phase compositions of the CoCrAlY alloys measured by EPMA (at.%).

T (K)	Ref.	B2			γ			σ			A2		
		Al	Cr	Co	Al	Cr	Co	Al	Cr	Co	Al	Cr	Co
1173	A4	36.6	13.2	50.3	5.6	34.3	60.1	2.9	54.0	43.1			
	A5	36.3	*13*.*6*	50.1	5.5	34.8	59.7	2.9	54.5	42.6			
	A6	36.3	14.2	49.5	5.5	35.3	59.2	2.9	54.8	42.3			
	A7	36.8	14.6	48.7				2.9	57.4	39.7			
1273	A4	32.0	18.1	49.9	6.6	35.8	57.6	4.0	53.5	42.5			
	A5	31.2	19.2	49.7	6.8	36.2	57.0	4.0	53.8	42.2			
	A6	31.7	18.9	49.5	6.7	36.2	57.1	3.9	54.1	42.0			
	A7	33.1	18.5	48.3				3.8	56.8	39.4			
1373	A4	28.2	20.7	51.1	8.4	33.7	57.9						
	A5	26.0	23.6	50.3	8.4	34.5	57.2						
	A6	16.2	36.9	46.8	8.0	36.5	55.5				12.4	41.6	46.0
	A7	22.3	33.4	44.2							11.7	48.4	39.9

The present experimental results confirm and refine the phase relationships originally documented by Ishikawa et al. [13]. Between 1273 and 1373 K, the B2+γ+σ three-phase region is replaced by an A2+B2+γ triangle, and the solubility of Cr in B2 drastically increases. Both are due to the shrinkage of the A2/B2 miscibility gap as the temperature increases, and to the associated dissolution of σ into A2. Ishikawa et al. [13] observed the A2/B2 order-disorder transition at 1473 K. The present measurements show very close compositions for B2 and A2 in equilibrium with γ at 1373 K and therefore, indicate that the miscibility gap is closed slightly above 1373 K.

### First-principles results

To provide a benchmark for the first-principles methodology used in the present work, fundamental properties of Al, Co, Cr and relevant phases, shown in Table 2, are compared with previous first-principles calculations [53,54] and data from various experiments [55–59]. Good agreement is seen for these properties for all three elements in their standard element reference (SER) state (pure, most stable at 298 K and 1 bar) [22]. It should be noted that Cr takes on an anti-ferromagnetic state at 0 K. The results agree with previous calculations and experiments [56,59,60] for anti-ferromagnetic Cr. The lattice parameters and bulk modulus of B2-(Al)(Co) agree with previous results [61,62] and hence, the predicted values for B2-(Co)(Cr) and B2-(Cr)(Al) can be adopted with confidence. No measured bulk moduli for σ-CoCr could be found in the literature and hence, only experimental lattice parameters are compared. The calculated values for the end-members σ-(Co)_8_(Co)_18_(Cr)_4_ and σ-(Co)_8_(Cr)_18_(Cr)_4_ are compared to the lattice parameter of a σ-Co_13_Cr_17_ alloy and are in good agreement with measurements by Dickins et al. [55].

**Table 2:** Various properties of the Al, Co, Cr, and the end-members of B2 and σ.

Phase	*V*_*0*_ (Å^3^/atom)	*B*_*0*_ (GPa)	Scaling factor	Source
Al (fcc)	16.50	78	0.63	Present work
	17.08	65		DFT[54]
		72		Experiment[56]
		79 (0 K)		Experiment[57]
Co (hcp)	10.88	210	0.78	Present work
	11.07	204		DFT[54]
		191		Experiment[56]
		196 (0 K)		Experiment[58]
Cr (bcc)	11.59	176	0.88	Present work
	11.56	189		DFT[60]
	11.58	258		DFT[54]
		190		Experiment[56]
		192 (0 K)		Experiment [59]
**B2 Phase**				
(Al)(Co)	11.61	178	0.87	Present work
		157		DFT[61]
		162±5		Experiment[61]
(Co)(Cr)	11.60	214	0.77	Present work
(Cr)(Al)	14.03	125	0.82	Present work
**σ Phase**				
(Al)_8_(Al)_18_(Cr)_4_	15.70	86	0.78	Present work
(Al)_8_(Cr)_18_(Cr)_4_	12.36	199	0.82	Present work
(Co)_8_(Al)_18_(Cr)_4_	13.07	141	0.78	Present work
(Al)_8_(Co)_18_(Cr)_4_	11.65	174	0.78	Present work
(Co)_8_(Co)_18_(Cr)_4_	11.12	197	0.69	Present work
(Co)_8_(Cr)_18_(Cr)_4_	11.33	249	0.75	Present work

These properties are derived from the energy vs. volume curves using the 4-parameter Birch-Murnaghan EOS. *B*_*0*_ denotes the bulk modulus. The bulk modulus at room temperature of Al, Co, and Cr are also presented, as reported by Kittel [56]. Other experimental temperatures are shown if known; reported 0 K values are extrapolated from low temperature data.

Results from our recent study on the Debye-Grüneisen model [63] indicate that using a calculated scaling factor for the Debye temperature is an accurate and efficient method to predict thermodynamic properties of pure elements and intermetallic phases in comparison with the more computationally demanding phonon supercell approach. Table 2 shows that the pure-element properties derived from the EOS fitting match experiments [56–58] and the DFT predictions [54,60]. Therefore, the Debye approximation is used to estimate the thermodynamic properties for all end-members of B2 and σ. Predicted enthalpies of formation (Δ_*f*_ *H*) for each end-member of B2 and σ are calculated at 298 K and are shown in Table 3. Note that the non-SER reference B2 formation energies are calculated with respect to the bcc phase of the pure elements and that σ energies are taken with respect to the fcc phase in the first sublattice, bcc in the second and bcc in the third following Ref. [7,17]. Experimental thermochemical data for B2 and σ end-members are unavailable at 298 K and consequently, only previous CALPHAD assessments and DFT results are compared in Table 3. Stein et al. [64] determined values of *Δ*_*f*_ *H*_*298*_ = -64.45 kJ/mol-atom and *Δ*_*f*_ *S*_*298*_ = -11.43 J/mol-atom of B2 based on modeling of experimental enthalpy data at 1100 K. The present DFT predictions using the scaling factor Debye-Grüneisen model are in excellent agreement with these values.

**Table 3:** Predicted enthalpies and entropies of formation of B2 and σ end-members at 298 K.

Phase	End-member	*Δ*_*f*_ *H*(kJ/mol-atom)	*Δ*_*f*_ *H (SER)*(kJ/mol-atom)	*Δ*_*f*_ *S* (J/mol-a)	*Δ*_*f*_ *S (SER)* (J/mol-a)	Source
(B2)	**(Al)(Co)**	**-66.89**	**-57.67**	-13.73	**-8.23**	Present work
Binary		-64.45		-11.43		CALPHAD[64]
	(Co)(Cr)	11.98	16.60	-1.73	1.03	Present work
	**(Cr)(Al)**	**-7.37**	**-2.78**	**-9.18**	**-6.45**	Present work
(σ)	**(Co)**_**8**_ **(Al)**_**18**_ **(Cr)**_**4**_	**-30.56**	**-24.58**	**-7.33**	**-4.05**	Present work
Ternary	**(Al)**_**8**_ **(Co)**_**18**_ **(Cr)**_**4**_	**-18.93**	**-13.39**	**-4.44**	**-1.13**	Present work
(σ)	(Co)_8_(Co)_18_(Cr)_4_	3.60	9.62	0.28	3.60	Present work
Binary	0 K		9.84			Present work
	0 K		11.65			DFT[65]
	(Co)_8_(Cr)_18_(Cr)_4_	5.72	6.19	0.16	0.16	Present work
	0 K		6.20			Present work
	0 K		8.39			DFT[65]
	(Al)_8_(Al)_18_(Cr)_4_	5.73	11.24	-6.02	-2.74	Present work
	(Al)_8_(Cr)_18_(Cr)_4_	1.84	1.84	-3.32	-3.32	Present work

Energies are shown in units of J/mol-formula and atom with the most stable end-members shown in bold text. Also, energies taken with respect to standard states are denoted with SER. Energies used for CALPHAD modeling are taken with different reference states depending on the sublattice models used. B2 formation energies are calculated with respect to the bcc phase of the pure elements. For σ, energies are taken with respect to the fcc phase in the first sublattice, bcc in the second and bcc in the third; same as the sublattice model implemented in the current work.

Unfortunately, calorimetric measurements for σ-CoCr have proven unreliable, as reported measured and calculated enthalpies of formation range from -3 to +10 kJ/mol-atom, shown in Ref. [65–67]. Also, the stability of σ in the Co-Cr system was suggested to range from 30 to 45 at.% Co, which is quite different from the calculated end-member compositions of 26.6 and 86.6% at.% Co. However, Downie and Arslan [67] measured the formation enthalpy of one σ–CoCr sample close to the composition of the end-member σ-(Co)_8_(Cr)_18_(Cr)_4_ at 473 K and found *Δ*_*f*_ *H* = 6.77 kJ/mol-atom. While no direct comparison can be made due to the composition and site fractions, the predicted value of *Δ*_*f*_ *H*_*298*_ = 6.19 kJ/mol-atom for σ-(Co)_8_(Cr)_18_(Cr)_4_ is in good agreement with the measured value. Table 3 shows the final values (non-SER columns) for B2 and σ that are used as input for the thermodynamic modeling in the present work.

**Fig 3:**
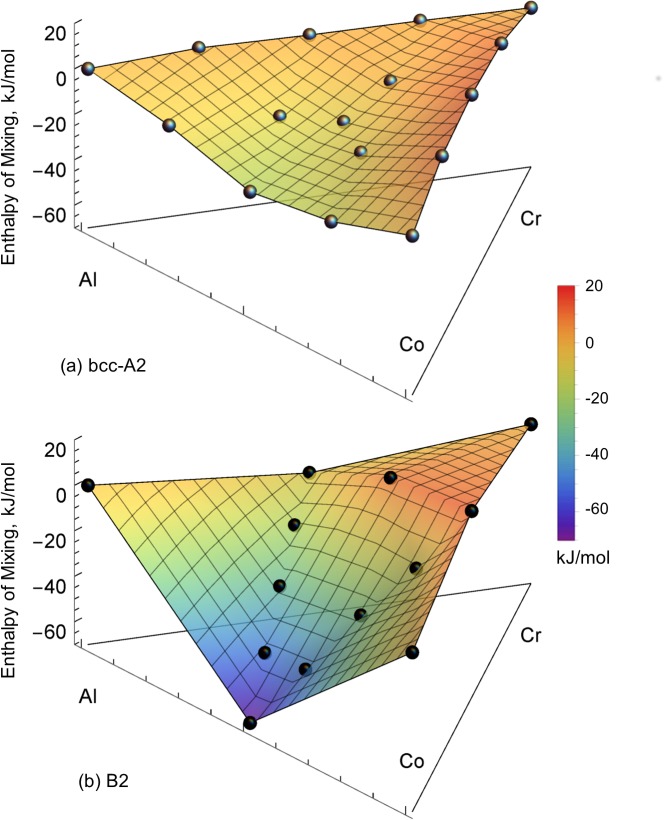
Predicted A2 (a) and B2 (b) enthalpies of mixing. These calculations are based on 16-atom binary and 32/36-atom ternary SQS supercells at 0 K. Grey points in (a) represent distinct A2 SQS compositions and black points in (b) represent B2 SQS compositions. A color map is added to guide the reader in viewing the energy surface.

For all binary and ternary bcc SQS calculations, RDFs indicate that the simultaneous relaxation of cell volume, shape and ion positions reduce the symmetry of the SQS cell environment. Additionally, ternary SQS calculations show symmetry reduction when only cell volume and shape are allowed to relax. Therefore, SQS supercells with the lowest energy and a coordination environment sufficiently close to the ideal bcc structure are used in the analysis. Calculated binary and ternary enthalpies of mixing are shown in Table 4 and Fig 3 for A2 and B2. Along with the symmetry, the magnetism of the SQS supercells is checked. Cobalt is ferromagnetic at 0 K with an average μB/atom of 2.2 while Al is non-magnetic. At 0 K, Cr is antiferromagnetic with no net average magnetic moment. Our calculations show that Al and Cr additions, both with no net magnetic moment, decrease the μB/atom of Co as a function of composition.

**Table 4:** Calculated enthalpies of mixing for solution A2 and B2.

Al	Co	Cr	SQS prototype	*ΔH*_*mix*_ (kJ/mol-atom)
***A2***				
**Al-Co**				
0.75	0.25		16	-8.71
0.5	0.5		16	-21.69
0.25	0.75		16	-18.57
**Co-Cr**				
	0.75	0.25	16	9.99
	0.5	0.5	16	11.98
	0.25	0.75	16	9.59
**Al-Cr**				
0.75		0.25	16	2.45
0.5		0.5	16	1.44
0.25		0.75	16	1.01
**Al-Co-Cr**				
0.5	0.25	0.25	32	-9.36
0.25	0.5	0.25	32	-6.86
0.25	0.25	0.5	32	-1.17
0.33	0.33	0.33	36	-7.86
**Al**	**Co**	**Cr**	**SQS prototype**	***ΔH***_***mix***_ **(kJ/mol-atom)**
***B2***				
**(Al)(Co)-(Co)(Cr) section**				
0.5	0.5	0		-67.75
0.375	0.5	0.125	32	-47.66
0.25	0.5	0.25	8	-27.20
0.125	0.5	0.375	32	-9.96
0	0.5	0.5		11.99
**(Co)(Cr)-(Cr)(Al) section**				
0	0.5	0.5		11.99
0.25	0.25	0.5	8	8.22
0.5	0	0.5		-7.96
**(Cr)(Al)-(Al)(Co) section**				
0.5	0	0.5		-7.96
0.5	0.125	0.375	32	-18.49
0.5	0.25	0.25	8	-32.77
0.5	0.375	0.125	32	-49.54
0.5	0.5	0		-67.75

These calculations are based on binary and ternary SQS calculations at 0 K with references taken as bcc-A2 for Al, Co, and Cr.

**Fig 4:**
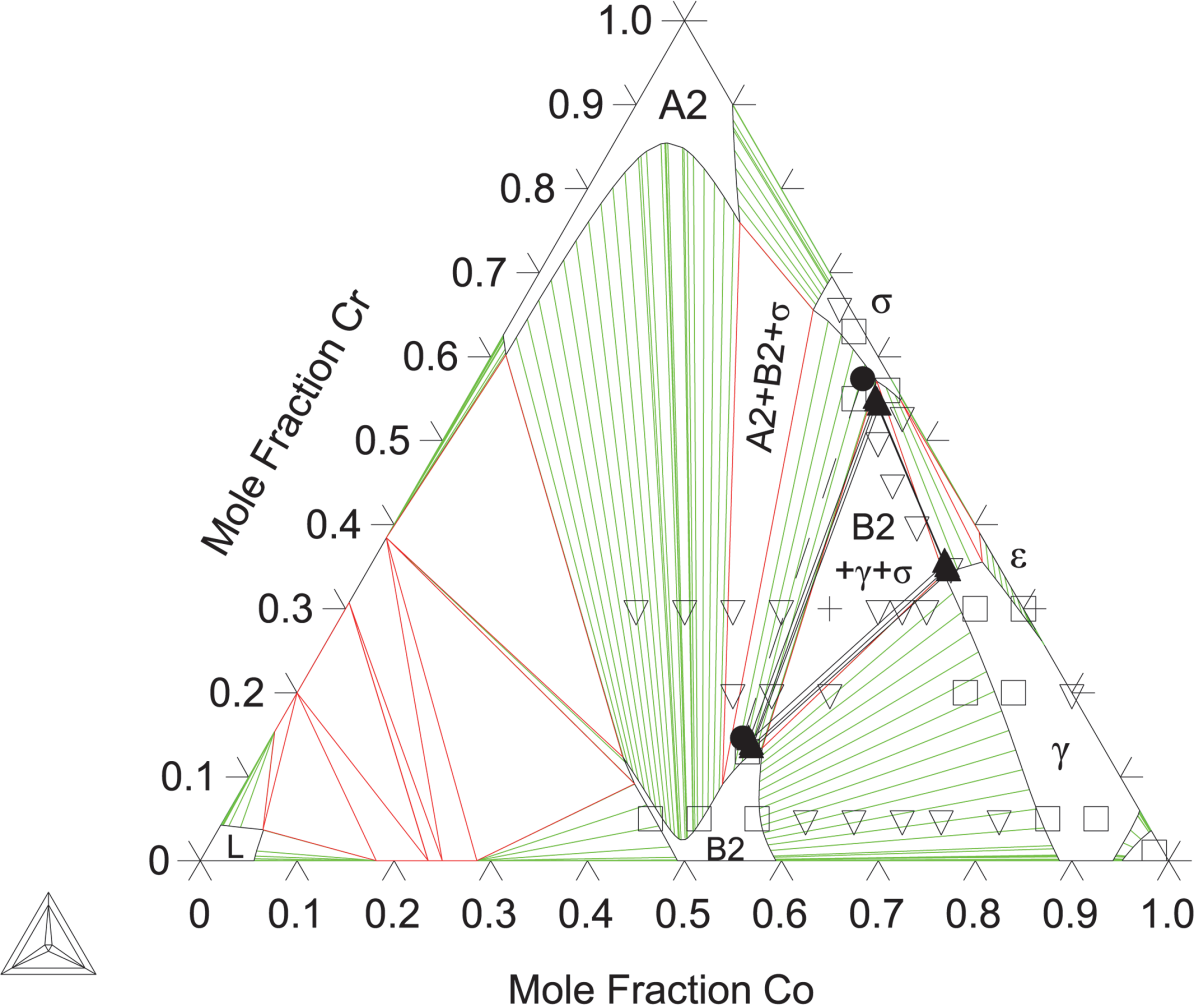
Al-Co-Cr Isothermal section at 1173 K. Shown with phase equilibria data from Moskvitina et al. [68]: single phase (◻), 2-phase (▿), and 3-phase (+). Phase equilibria data from the present work: 2-phase (●), 3-phase (▲).

The SQS calculations, shown in Fig 3 and Table 4, predict the formation of a low-temperature miscibility gap in both Co-Cr and Al-Cr while strong negative mixing is seen in Al-Co. These predictions agree well with experimental observations as well as previous models of the Al-Co [16,64] and Co-Cr [17] binary systems currently adopted for the thermodynamic modeling. In the Co-Cr binary modeled by Oikawa et al. [17], a low temperature A2 miscibility gap is seen at 298 K. Furthermore, the predicted A2 Al_0.5_Co_0.5_ mixing enthalpy (Table 3) is in good agreement with the Al-Co assessment [16]. However, the Al-Cr model [20] does not produce a low-temperature A2 miscibility gap and hence, disagrees with our current predictions. This is due to a lack of low-temperature themochemical data available in the literature at the time when Al-Cr was originally modeled. Ternary A2 miscibility gaps are also predicted (Fig 3A), especially along the Al_0.5_Co_0.5_-Cr cross-section where the composition Al_0.5_Co_0.5_ shows the lowest mixing enthalpy on the whole energy surface. A convex energy surface would produce a tangent from Al_0.5_Co_0.5_ to pure Cr that generates a large miscibility gap across the entire Al_0.5_Co_0.5_-Cr cross-section. When B2 mixing is also taken into account, as shown in Fig 3B, this A2 and B2 miscibility gap becomes more prominent across the Al_0.5_Co_0.5_-Cr cross-section in accordance with the experiments by Ishikawa et al. [13]. The extremely high formation energy of the end-member (Al)(Co) is responsible for this behavior. Additionally, the SQS results show that A2 is more stable than B2 near Cr because *ΔH*_*mix*_ = -1.17 kJ/atom for A2 at the composition Al_0.25_Co_0.25_Cr_0.5_ while *ΔH*_*mix*_ = 8.22 kJ/atom for B2 at the same composition. These SQS results are in good agreement with measured phase compositions by Ishikawa et al. [13] as well as the present experimental findings. As shown later in Fig 4, this A2/B2 miscibility gap is still observed at 1173 K.

### CALPHAD modeling results

The three binary models, Al-Co [16], Co-Cr [17], and Al-Cr [20], are extrapolated to the ternary system using the partitioning model [26]. In addition, thermodynamic parameters from DFT, listed in Table 3, are used as input. The binary B2-(Al)(Co), B2-(Cr)(Al), σ-(Co)_8_(Co)_18_(Cr)_4_ and σ-(Co)_8_(Cr)_18_(Cr)_4_ end-members are fixed by previous models and cannot be modified without reassessment of those systems. The initial ternary extrapolation produces a phase diagram with satisfactory phase boundaries for fcc, A2, and B2 at 1173 K compared to experiments, but is at variance with the experimental isothermal sections at all other temperatures [13,68]. A2/B2 regions do not separate at the order/disorder compositions even at temperatures as high as 1623 K, which is at significant variance with experiments (see experimental Section 6.1 for discussion). The poor extrapolation is a result of fixing the B2-(Cr)(Al) end-member to the value proposed by Dupin et al. [7] in their modeling of the Al-Cr-Ni ternary system. Dupin et al. [7] assessed *Δ*_*f*_ *H* of B2-(Cr)(Al) to be -13.719 kJ/mol-atom, which is much more stable than the value of -7.37 kJ/mol-atom predicted by DFT in the present work. However, modifying this energy will require significant reassessment of Al-Cr-Ni ternary description and is outside the scope of this work. As a consequence of this fixed B2 end-member, additional interaction parameters for the B2 phase are needed to correctly reproduce its miscibility gap.

**Fig 5:**
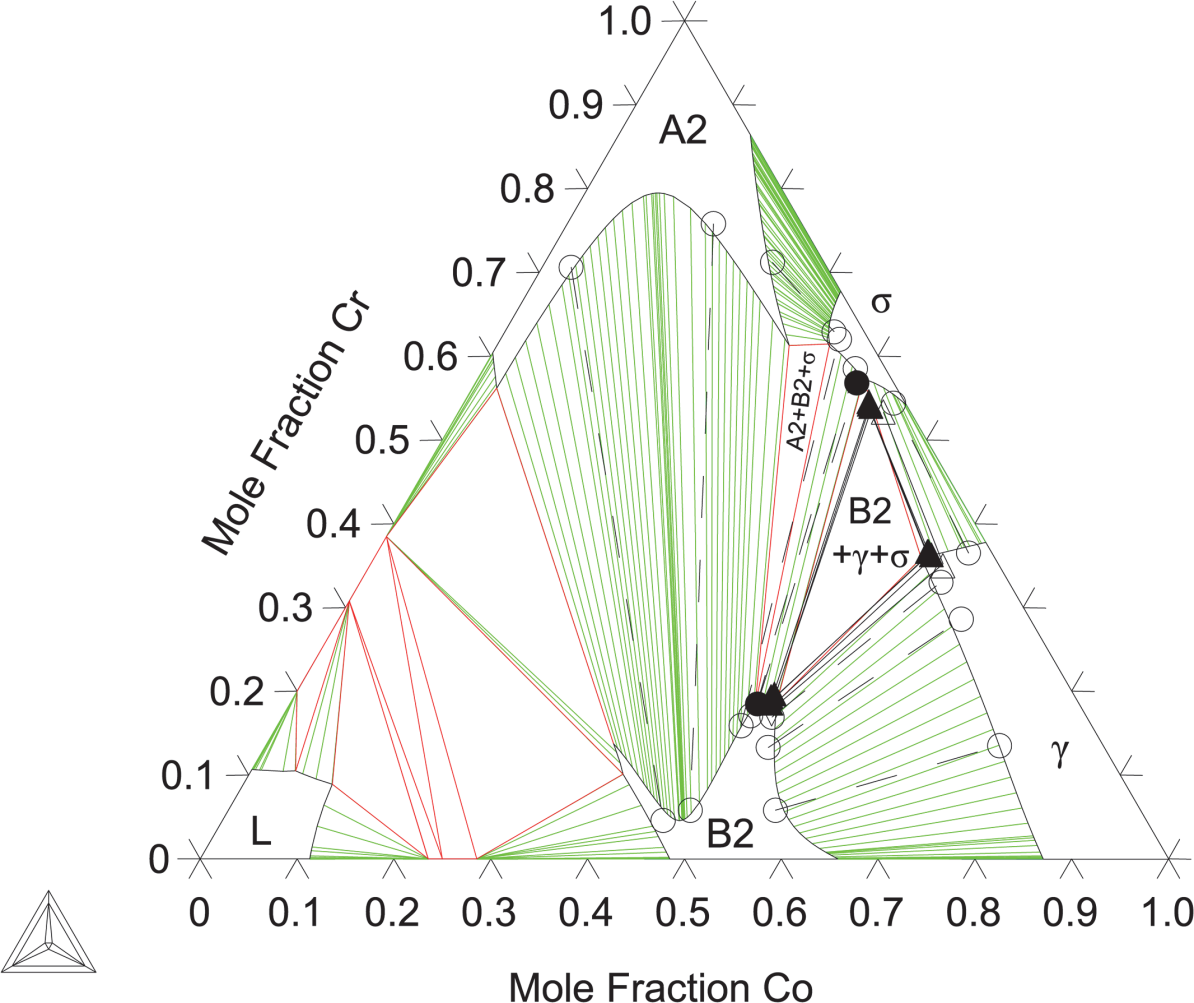
Al-Co-Cr Isothermal section at 1273 K. Shown with phase equilibria data from Ishikawa et al. [13]: 2-phase (엯), and 3-phase (▿). Experimental phase equilibria data from the present work: 2-phase (●), 3-phase (▲).

**Fig 6:**
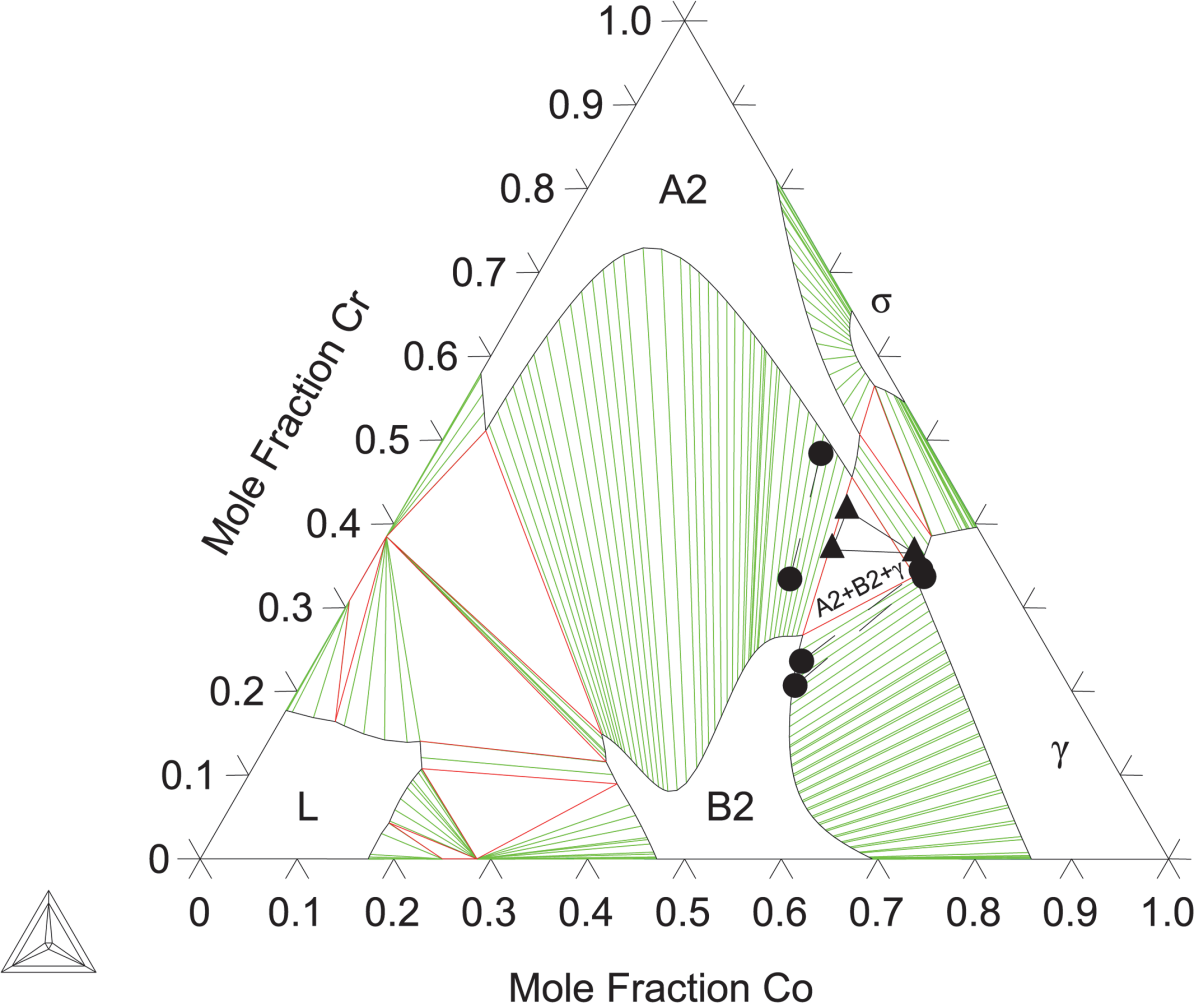
Al-Co-Cr Isothermal section at 1373 K. Shown with phase equilibria data from the present work: 2-phase (●), 3-phase (▲).

**Fig 7:**
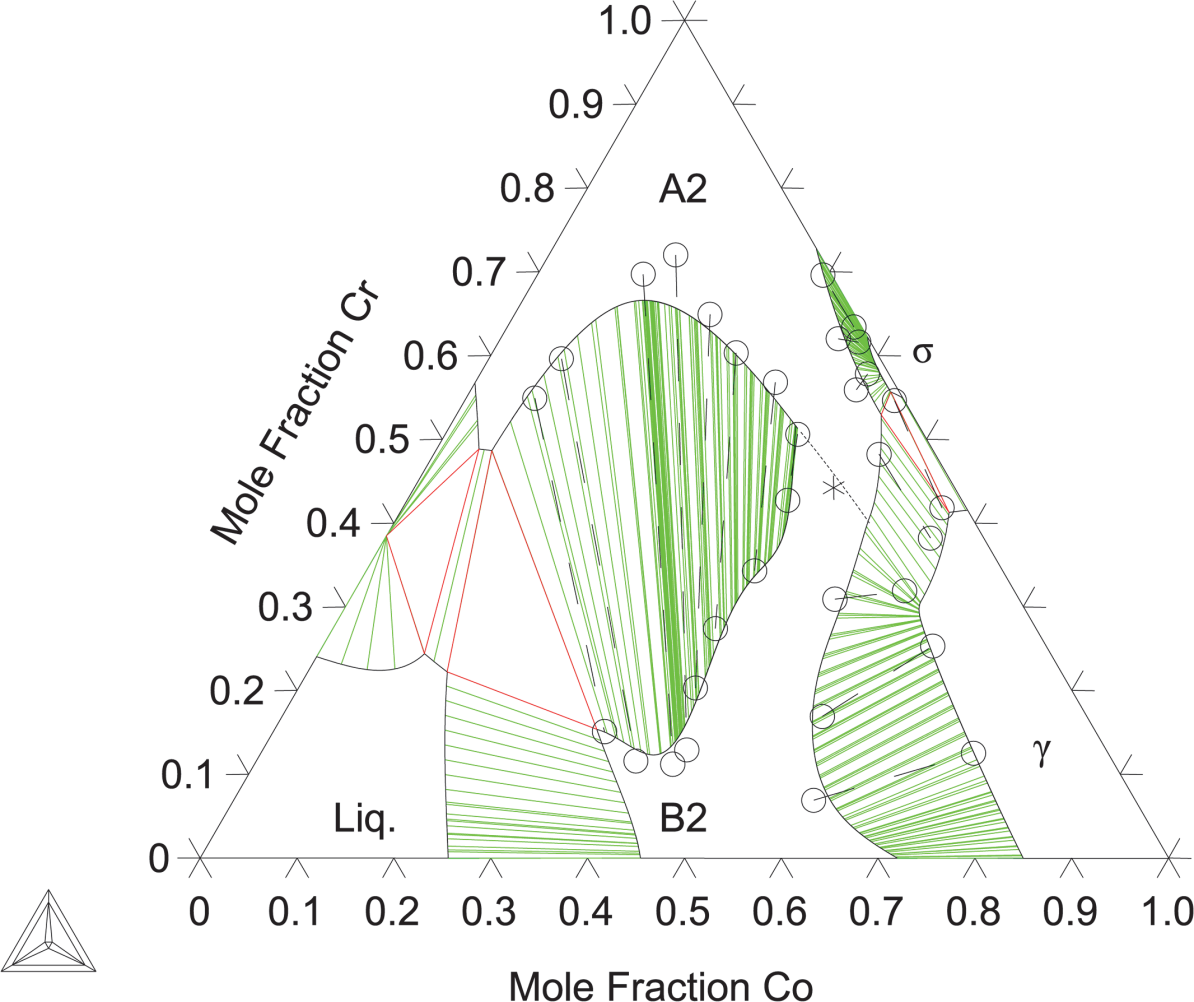
Al-Co-Cr isothermal section at 1473 K. Phase equilibria data from Ishikawa et al. [13]: 2-phase (엯), order-disorder transition (*). The calculated order-disorder transition is shown with (∙ ∙ ∙).

**Fig 8:**
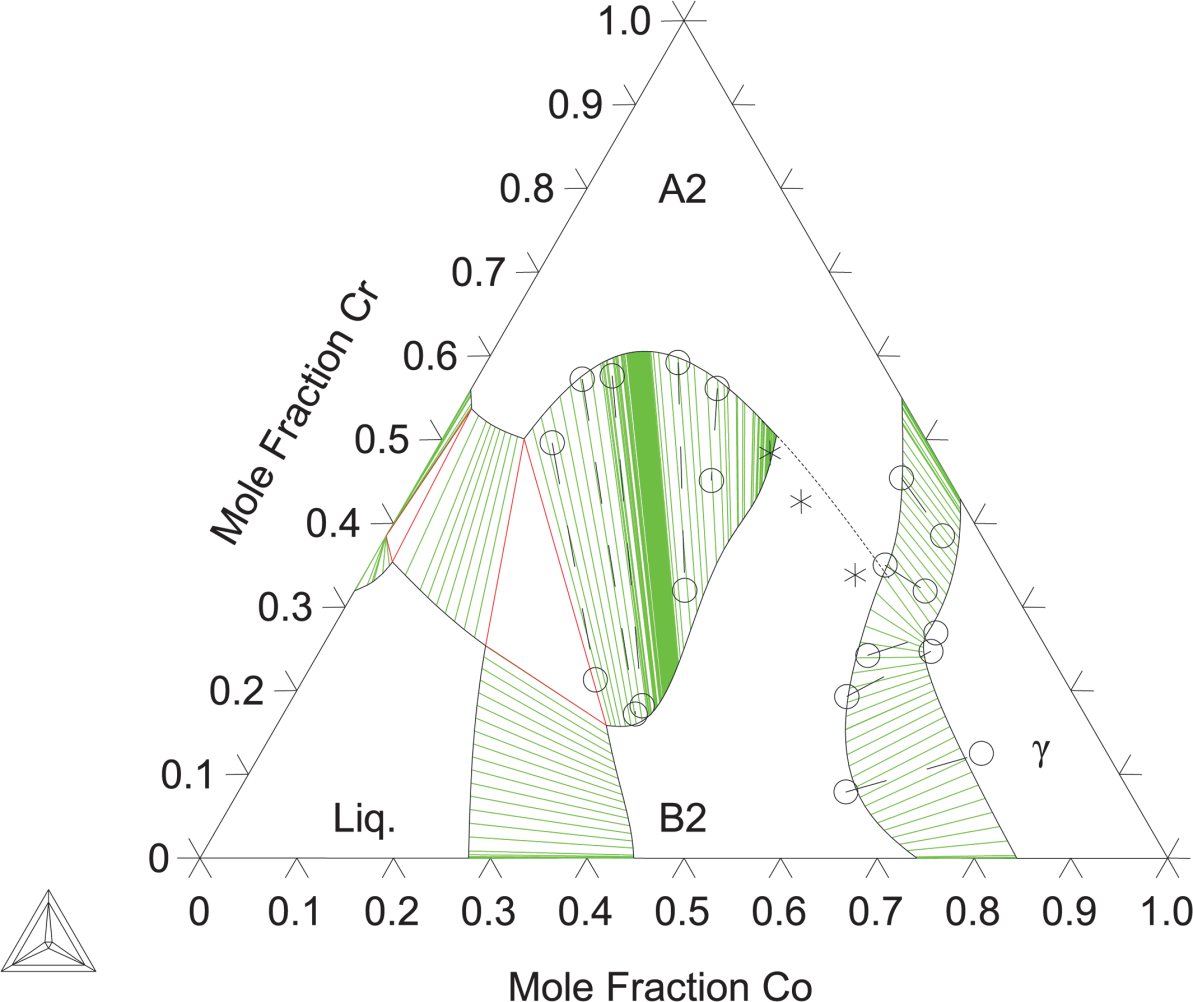
Al-Co-Cr Isothermal section at 1573 K. Phase equilibria data from Ishikawa et al. [13]: 2-phase (엯), order-disorder transition (*). The calculated order-disorder transition is shown with (∙ ∙ ∙).

The binary B2-(Co)(Cr) end-member and the Co-Cr interaction parameters are evaluated by fitting to the present EPMA results at 1173, 1273, and 1373 K (Table 1) data from Ishikawa et al. [13] as well as first-principles thermochemical data. Given the complexity of the A2/B2 miscibility gap region, as seen in Figs 4–8, ternary, temperature dependent interaction parameters are needed to describe the B2-(Al)(Co) extension into the ternary as well as the A2/B2 transition temperatures. Interaction parameters for the A2 are also needed to capture the Al and Co solubility. The evaluated parameters are shown in Table 5 and built into a thermodynamic database, which can be found in S1 Dataset.

**Table 5:** Model parameters and functions for the ternary Al-Co-Cr system.

Phase	Parameters	Values	References
**liquid (L) phase: (Al,Co,Cr)**_**1**_	${}^{0}L_{Al,Co,Cr}^{Liq}$	+30000	Present work
**fcc (γ) phase: (Al,Co,Cr)**_**1**_	${}^{0}L_{Al,Co,Cr}^{fcc}$	+17295	Present work
**hcp (ε) phase: (Al,Co,Cr)**_**1**_	${}^{0}L_{Al,Co,Cr}^{hcp}$	+25000	Present work
**bcc (A2) phase: (Al,Co,Cr,Va)**_**1**_	${}^{0}L_{Al,Co}^{A2}$	+*GB*2*ALCO*−*LB*2*ALCO*	Present work
	${}^{0}L_{Al,Cr}^{A2}$	−54900 + 10 ×*T*	*COST 507*[20]
	${}^{0}L_{Co,Cr}^{A2}$	+1033−1.481 × *T*	*Oikawa et al*.[17]
	${}^{1}L_{Co,Cr}^{A2}$	+11972−13.374 × *T*	*Oikawa et al*.[17]
	${}^{0}L_{Al,Va}^{A2}$	+*GB*2*ALVA*−*LB*2*ALVA*	*Dupin et al*.[7]
	${}^{0}L_{Co,Va}^{A2}$	+*GB*2*COVA*−*LB*2*COVA*	Present work
	${}^{0}L_{Cr,Va}^{A2}$	+100000	*Dupin et al*.[7]
	${}^{0}L_{Al,Co,Cr}^{A2}$	−*LB*2*ALCO* + *LBALCOMCR* +*LBCOCRMAL*+*L*1*BCRALMCO*	Present work
	${}^{1}L_{Al,Co,Cr}^{A2}$	+*LALCO*2*CR*−*LB*2*ALCO* +*LBALCOMCR* + *LBCOCRMAL*	Present work
	${}^{2}L_{Al,Co,Cr}^{A2}$	−*LB*2*ALCO* + *LBALCOMCR* +*LBCOCRMAL*−*L*1*BCRALMCO*	Present work
**beta (B2) phase: (Al,Co,Cr,Va)**_**0.5**_ **(Al,Co,Cr,Va)**_**0.5**_	${}^{0}G_{Al:Co}^{B2}={}^{0}G_{Co:Al}^{B2}$	+0.5 × *GB*2*ALCO*−0.5 × *LB*2*ALCO*	Present work
	$TC_{Al:Co}^{B2}=TC_{Co:Al}^{B2}$	-1450	Present work
	$BMAG_{Al:Co}^{B2}=BMAG_{Co:Al}^{B2}$	-1.35	Present work
	${}^{0}G_{Co:Cr}^{B2}={}^{0}G_{Cr:Co}^{B2}$	+0.5 × *GB*2*COCR*	Present work
	${}^{0}G_{Cr:Al}^{B2}={}^{0}G_{Al:Cr}^{B2}$	+0.5 × *GB*2*CRAL*	Present work
	${}^{0}G_{Al:Va}^{B2}={}^{0}G_{Va:Al}^{B2}$	+0.5 × *GB*2*ALVA*−0.5 × *LB*2*ALVA*	Present work
	${}^{0}G_{Co:Va}^{B2}={}^{0}G_{Va:Co}^{B2}$	+0.5 × *GB*2*COVA*−0.5 × *LB*2*COVA*	Present work
	${}^{0}G_{Cr:Va}^{B2}={}^{0}G_{Va:Cr}^{B2}$	0	Present work
	${}^{0}L_{Co,Cr:Al}^{B2}={}^{0}L_{Al:Co,Cr}^{B2}$	+0.5 × *LBCOCRMAL*	Present work
	${}^{0}L_{Al,Cr:Co}^{B2}={}^{0}L_{Co:Al,Cr}^{B2}$	+0.5 × *LBCRALMCO*	Present work
	${}^{0}L_{Al,Co:Cr}^{B2}={}^{0}L_{Cr:Al,Co}^{B2}$	+0.5 × *LBALCOMCR* + *BU*1*ALCO*	Present work
	${}^{1}L_{Co,Cr:Al}^{B2}={}^{1}L_{Al:Co,Cr}^{B2}$	+0.5 × *L*1*BCOCRMAL*	Present work
	${}^{1}L_{Al,Cr:Co}^{B2}={}^{1}L_{Co:Al,Cr}^{B2}$	+0.5 × *L*1*BCRALMCO*	Present work
	${}^{1}L_{Al,Co:Cr}^{B2}={}^{1}L_{Cr:Al,Co}^{B2}$	+0.5 × *L*1*BALCOMCR*	Present work
	${}^{0}L_{Al,Co,Cr:Al}^{B2}={}^{0}L_{Al:Al,Co,Cr}^{B2}$	+0.5 × *LAL*2*COCR*	Present work
	${}^{0}L_{Al,Co,Cr:Co}^{B2}={}^{0}L_{Co:Al,Co,Cr}^{B2}$	+0.5 × *LALCO*2*CR*	Present work
	${}^{0}L_{Al,Co,Cr:Cr}^{B2}={}^{0}L_{Cr:Al,Co,Cr}^{B2}$	+0.5 × *LALCOCR*2	Present work
**sigma (σ) phase: (Al,Co)**_**8**_ **(Al,Co,Cr)**_**18**_ **(Cr)**_**4**_	${}^{0}G_{Al:Al:Cr}^{\sigma }$	+161148	Present work
	${}^{0}G_{Al:Cr:Cr}^{\sigma }$	+47886	Present work
	${}^{0}G_{Co:Co:Cr}^{\sigma }$	−16899−29.814 × *T*	*Oikawa et al*.[17]
	${}^{0}G_{Co:Cr:Cr}^{\sigma }$	−259935 + 85.097 × *T*	*Oikawa et al*.[17]
	${}^{0}G_{Al:Co:Cr}^{\sigma }$	−617537	Present work
	${}^{0}G_{Co:Al:Cr}^{\sigma }$	−931862	Present work
	${}^{0}L_{Co:Al,Cr:Cr}^{\sigma }$	−195992	Present work
**Function**	**Value**		
***LAL*2*COCR***	0		Present work
***LALCO*2*CR***	−78970 + 89.123 × *T*		Present work
***LALCOCR*2**	0		Present work
***LBCOCRMAL***	28320−16.474 × *T*		Present work
***LBCRALMCO***	0		Present work
***LBALCOMCR***	−46432		Present work
***L*1*BCOCRMAL***	0		Present work
***L*1*BCRALMCO***	13276		Present work
***L*1*BALCOMCR***	0		Present work
***GB*2*ALCO***	−138500 + 34.620 × *T*		Dupin and Ansara[16]
***LB*2*ALCO***	54531−37.04 × *T*		Present work
***GB*2*COCR***	35909−16.474 × *T*		Present work
***GB*2*CRAL***	−4000		*Dupin et al*.[7]
***BU*1*XY*** (X,Y = Al,Co,Cr)	−0.5 × *LB*2*XY*		Present work

Only A2, B2, and σ binary parameters are listed in full for their importance, all other binary parameters can be found in the respective binary Al-Co[16], Co-Cr[17], and Al-Cr[20] references as well as the attached database file. Parameters are in units of J/mol-formula.

Fig 4 shows the calculated isothermal section at 1173 K compared with the present EPMA results as well as experimental data from Moskvitina et al. [68]. A good agreement between calculations and experiments is reached. Fig 5 shows the calculated isothermal section at 1273 K, which agrees very well with the present experiments as well as measurements by Ishikawa et al. [13]. Fig 6 shows a calculated isothermal section at 1373 K, which agrees well with our experiments, except for the B2 composition in the A2+B2+γ three phase tie-triangle and the A2-B2 tie-line. This is attributed to the fact that the A2/B2 miscibility gap disappears very rapidly with increasing temperature, and as a consequence the morphology of this region of the diagram is extremely sensitive to temperature variations around 1373 K. The A2+B2+γ three phase triangle is predicted to disappear at 1423 K measurements by Ishikawa et al. [13] at 1473 K. Fig 7 plots the 1473 K isothermal section, which fits experimental measurements from Ishikawa et al. [13] quite well. At this temperature, the A2/B2 miscibility gap is replaced by a second-order transition represented by a dotted line. The calculation near the transition region shows that the B2 ordering gravitates towards the disordered composition with more Cr additions on the Co-Cr rich side of the phase diagram. This is in good agreement with the experimental results on the order/disorder alloys found by Ishikawa et al. [13]. Fig 8 shows the 1573 K isothermal sections. Furthermore, the A2/B2 and B2/γ tie-lines as well as A2/B2 transition points show good agreement at 1573 and 1623 K compared with data from Ishikawa et al. [13] (not included here). An overall good thermodynamic description of Al-Co-Cr, which takes into account predicted first-principles thermochemical data, is produced in this work. We have demonstrated a model that is able to reproduce the rapid replacement of one three-phase σ+B2+γ tie-triangle by another and the sharp increase in the solubility of Cr in B2 from 1273 to 1373 K, as observed experimentally (see Section 6.1).

## Conclusions

The Al-Co-Cr system is investigated thoroughly using first-principles calculations, XRD, and EPMA measurements to produce a complete CALPHAD thermodynamic description. First-principles DFT calculations predict a large A2/B2 miscibility gap in the ternary system, which is demonstrated experimentally using phase composition measurements from 1173 to 1373 K. In addition, the experimentally-measured phase compositions for A2, B2, γ and σ are in good agreement with previous experimental results [13]. To aid in the modeling of the ternary system, the Debye-Grüneisen model is used to predict finite-temperature data of B2 and σ such as heat capacities, entropies, and enthalpies. It is found that the complex A2/B2 phase region, which includes an order-disorder transition, can be accurately described with a partitioned bcc model. The calculated B2 ordering compositions are also shown to be in agreement with previous as well as present experimental studies. Overall, a consistent thermodynamic description of the Al-Co-Cr system is produced and its accuracy for predicting thermodynamic properties of all phases relevant for MCrAl-base coatings is confirmed.

## Supporting Information

S1 Dataset Thermodynamic database for the Al-Co-Cr system. This dataset contains the models and parameters built using Thermo-Calc for the Al-Co-Cr alloy system in text format.
(TXT)
